# New Biofunctional Loading of Natural Antimicrobial Agent in Biodegradable Polymeric Films for Biomedical Applications

**DOI:** 10.1155/2016/6964938

**Published:** 2016-11-14

**Authors:** Bakhtawar Ghafoor, Murtaza Najabat Ali, Umar Ansari, Muhammad Faraz Bhatti, Mariam Mir, Hafsah Akhtar, Fatima Darakhshan

**Affiliations:** Biomedical Engineering and Sciences Department, School of Mechanical and Manufacturing Engineering (SMME), National University of Sciences and Technology (NUST), Islamabad, Pakistan

## Abstract

The study focuses on the development of novel* Aloe vera* based polymeric composite films and antimicrobial suture coatings. Polyvinyl alcohol (PVA), a synthetic biocompatible and biodegradable polymer, was combined with* Aloe vera*, a natural herb used for soothing burning effects and cosmetic purposes. The properties of these two materials were combined together to get additional benefits such as wound healing and prevention of surgical site infections. PVA and* Aloe vera* were mixed in a fixed quantity to produce polymer based films. The films were screened for antibacterial and antifungal activity against bacterial (*E. coli*,* P. aeruginosa*) and fungal strains (*Aspergillus flavus* and* Aspergillus tubingensis*) screened.* Aloe vera* based PVA films showed antimicrobial activity against all the strains; the lowest* Aloe vera* concentration (5%) showed the highest activity against all the strains.* In vitro* degradation and release profile of these films was also evaluated. The coating for sutures was prepared,* in vitro* antibacterial tests of these coated sutures were carried out, and later on* in vivo* studies of these coated sutures were also performed. The results showed that sutures coated with* Aloe vera*/PVA coating solution have antibacterial effects and thus have the potential to be used in the prevention of surgical site infections and* Aloe vera*/PVA based films have the potential to be used for wound healing purposes.

## 1. Introduction

Nosocomial infections are hospital-acquired infections (HAI) that usually develop in patients during their hospital stay, affecting the health expenditure of the patient [1]. The main factors that make patients prone to nosocomial infection include concurrent infections, medical devices, surgery, immunosuppressive agents, and emergence of multidrug resistant pathogens. Pathogens are responsible for such infections known as nosocomial pathogens. Among them 90% bacterial pathogens are involved; however mycobacterial, viral, fungal, or protozoal agents are less commonly involved [2]. According to the data,* Escherichia coli*,* Staphylococcus aureus*, enterococci, and Pseudomonas* aeruginosa* are the most common nosocomial pathogens [3]. Among the fungal pathogens,* Candida albicans* [4],* Aspergillus* spp., and especially* Aspergillus fumigatus*,* A. flavus*, and* A. terreus* have also been reported as the common cause of nosocomial infection in highly immunocompromised patients. These pathogens can be transmitted through either inhalation or direct contact with occlusive materials [5, 6].

One of the reasons of nosocomial infections is surgical site infections (SSIs) mainly caused due to infected suture materials used in surgery and medical implants [7]. These infections are usually difficult to resolve and may cause complications in extreme cases. In order to prevent surgical site infections, scientists have been using several natural and synthetic materials like plant extracts and polymers which may be used as coating materials on surface of medical devices such as surgical implants or sutures [8]. The addition of antibiotics to these coating biomaterials can provide the local delivery of antibiotic directly at implantation or suture site, thereby decreasing the onset of infection [9]. Synthetic and natural biomaterials have also been used in other biomedical applications such as drug delivery systems, wound infections, and antitumor and anti-inflammatory agents [10].

Among synthetic biomaterials, one of the extensively used polymers is poly(vinyl alcohol) (PVA). Due to its suitable chemical and physical properties, biocompatibility, biodegradability, easy preparation with excellent film forming properties, and nontoxic nature, PVA has been studied intensively in different biomedical applications including wound dressings, contact lenses, coatings for sutures, and catheters [11, 12].

*Aloe vera*, as a natural source of bioactive compounds, is widely studied for biomedical applications.* Aloe vera* belongs to the Liliaceae family and is known as the oldest therapeutic herb. It has the ability to promote wound healing as well as treat burnt areas on the skin [13, 14]. Due to its properties, many researchers have shown the antibacterial, antiviral, antitumor, and anti-inflammatory activity of different parts of* Aloe vera* such as its stem, root, and leaf extracts [15–17]. The chemical composition of* Aloe vera* has also proved its potential use in cosmetic formulations, food supplements, and medical devices [15, 18, 19].

The inner part of* Aloe vera* contains a clear mucilaginous tissue commonly known as* Aloe* gel. The major portion of* Aloe* gel contains water while almost 1% contains bioactive compounds such asaloin, emodin (anthraquinones), flavonoids, saponin, and* Aloe*-mannan along with many different amino acids and vitamins. These bioactive compounds play a major role for antibacterial activity of* Aloe* gel [15, 20, 21].

The present work focuses on the antibacterial and antifungal activity of* Aloe vera*/PVA composite membranes and the application of these blends in the prevention of nosocomial infections; for the specific purpose of investing this, sutures coated with the PVA/*Aloe* gel blend have been used for both* in vitro* and* in vivo analysis*.* Aloe vera*/PVA films have been characterized through SEM and FTIR analysis. The results of* in vitro* and* in vivo* analysis proposed that this composition can be used as a coating for the prevention of surgical site infections that are caused by infections through sutures. To our knowledge, such biofunctional* Aloe* loaded PVA coatings have not been investigated against surgical infection causing bacteria in recent studies. The results of our study indicate the potential of such coatings as part of larger preventive measures against surgical infections.

## 2. Materials and Methods

### 2.1. Collection of Plant Material

Fresh* Aloe vera* plants were collected from local nurseries and the leaves washed well with distilled water to remove all contaminants present at the surface. The gel was harvested from the leaves in an autoclaved container and kept at room temperature for further use; the storage time of the gels at room temperature was one minute. During this time period, any residual solid leaf particles were mechanically separated from the gel.

### 2.2. Test Organisms for* In Vitro* Studies

In order to investigate antimicrobial and antifungal activity (*in vitro* studies), pure cultures of bacterial and fungal strains including* Pseudomonas aeruginosa (P. aeruginosa)*,* Escherichia coli (E. coli)*,* Aspergillus tubingensis,* and* Aspergillus flavus* were obtained from Mycovirus Research Lab, National University of Sciences and Technology (NUST) H-12, Islamabad. The pure bacterial and fungal cultures were stored in nutrient agar at 4°C.

### 2.3. Suture Materials

Commercially available silk braided black surgical sutures, nonabsorbable (1.5 metric, size 4-0) supplied by Foosin Medical supplies Inc., Ltd., Shandong, China and manufactured by WEGOSUTURES, were used to carry out* in vitro* and* in vivo* studies. The suture material was delivered in sterile single peelable foil packages and stored at room temperature. For investigation, the sutures were cut into defined lengths (1 cm) under aseptic conditions.

### 2.4. Preparation of* Aloe vera* Based PVA Films

Polyvinyl alcohol (PVA) supplied by AppliChem, Germany, a biocompatible polymer, was used for the formation of polymer/*Aloe vera* films [22]. Dimethyl Formamide (DMF) manufactured by TEDIA Company Inc, USA, was selected as solvent for the formation of PVA-*Aloe vera* films, due to its high volatility.

Solvent-casting method was used for the fabrication of* Aloe vera* gel/PVA films. 1 g of PVA was dissolved in 40 mL of DMF. The solution was stirred with a constant RPM of 580 at 60°C until PVA was completely dissolved, and a clear solution was obtained. This was followed by the addition of different amounts of* Aloe* gel.* Aloe* gel was added in the amounts of 5%, 10%, 15%, and 20%, respectively, for the fabrication of* Aloe vera*/PVA films with varying* Aloe* gel compositions. The heating was turned off while constant magnetic stirring was continued to obtain a homogenized mixture of* Aloe vera* gel and PVA in DMF. The mixture was poured into Petri dishes and placed in oven at 37°C for 20 h to evaporate the solvent completely and dry films were harvested for further testing. A solution of PVA in DMF was also prepared by the same procedure to obtain PVA films that were used as control for antimicrobial activity.

### 2.5. Antimicrobial Testing of* Aloe vera* Based PVA Films

The antifungal and antibacterial activities of films were evaluated using standard procedure of disc diffusion [23]. For antibacterial activity sterile nutrient agar (pH: 7.4) was prepared using Tryptone 10 g supplied by BioWorld, USA, yeast extract 5 g, supplied by MERCK, Germany, Sodium Chloride 10 g supplied by AnalaR, England, and nutrient agar 12 g supplied by MERCK, Germany, dissolved in 1000 mL of distilled water. After autoclaving the nutrient agar was poured in Petri dishes which were inoculated with the 0.1 mL of bacterial inoculum from preculture of test bacterial strains.

For antifungal investigation, sterile potato dextrose agar was prepared and poured onto the Petri plates and pure fungal cultures were obtained from test fungal strains.

For disc diffusion test, films were cut into discs of about 7 mm in diameter and placed on the bacterial and fungal inoculated plates with certain distances. Each Petri plate contained six discs one of which included the control sterile Whatman filter paper number 1, PVA film, and other four* Aloe vera*/polymer based films with varying concentrations of* Aloe vera* (5%, 10%, 15%, and 20%). Antimicrobial activity of pure* Aloe vera* gel was also recorded using well diffusion method. 10 mm of diameter of well was made in solid agar medium in which 0.1 mL of pure* Aloe vera* gel was delivered into the well after incubating the plate with the bacterial strains.

For antibacterial testing a positive control (Tetracycline disc) was used. All plates were incubated at 37°C for 24 h. The zone of inhibition diameter in millimeter (mm) was measured. The study was performed in triplicate and mean was calculated.

### 2.6. Characterization of* Aloe vera* Based PVA Films

#### 2.6.1. Fourier Transform Infrared (FTIR) Analysis

Fourier transform infrared (FTIR) spectroscopy (Perkin Elmer, spectrum 100 FTIR spectrophotometer) of* Aloe vera*/PVA films was carried out (at 256 scans, 8 cm^−1^ resolution) to investigate the presence of functional groups and types of interaction between the* Aloe vera* and PVA components.

#### 2.6.2. Morphological Analysis: SEM

Scanning Electron Microscopy (SEM) was performed to find out the surface morphology of the casted films. The assessment of the surface morphology of the* Aloe vera*/PVA based films was done using JSM-6490A Analytical scanning electron microscope (JEOL, Tokyo, Japan). SEM images were collected at an activation voltage of 20 kV.

### 2.7. *In Vitro* Degradation and* Aloe* Release Profile Testing of* Aloe vera* Based PVA Film

The degradation profile was assessed by recording weight differences after regular time intervals while* Aloe* release profile of* Aloe vera*/PVA films was assessed through UV-Vis spectrophotometry. A portion of* Aloe vera*/PVA films with measurable size (1′′ by 1′′) were cut and placed in 3 mL of PBS (pH 7.4) at 37°C. The remaining PBS was removed after every 10-minute interval and replaced with fresh 3 mL of PBS. The films were weighed before addition of PBS and afterwards they were taken out of the PBS solution, in wet state; the weights were subtracted and recorded. Moreover the drained PBS solutions were evaluated for* Aloe* release profile by UV-VIS spectrophotometer (Systronics 2202) absorbance at *λ*_max_ = 301 nm. The degradation and release tests were carried out in triplicate and an average value was calculated.

### 2.8. Coating for the Sutures

Dip coating method was used to coat the sutures. For dip coating, the solution was prepared by mixing 2 g of* Aloe vera* and 1 g of PVA in 40 mL of DMF. The sutures (30 cm length) were first sterilized and then dipped in the dip coating solution (for 60 minutes) followed by removal and air drying of suture for 24 h. The confirmation of coating of the suture was done by measuring the weight before coating and after coating.

### 2.9. *In Vitro* Evaluation of Coated and Uncoated Sutures

The silk sutures (with and without* Aloe vera*/PVA coating) were evaluated* in vitro* for antibacterial activity against two bacterial strains, that is,* E. coli* and* P. aeruginosa*. Nutrient agar media (pH: 7.4) plates were prepared and the coated suture of the size 4 cm was placed over agar. The plates were then inoculated with bacterial strains (*E. coli* and* P. aeruginosa*) and antibacterial activity was recorded.

### 2.10. *In Vivo* Evaluation of Coated Sutures

BALB/c mice were purchased from National Institute of Health (NIH) for the* in vivo* analysis of coated sutures. To check the antimicrobial activity of the coated sutures* in vivo*, mice were given an incision of about 2 cm on both sides of the spine. The incision was inoculated with* E. coli* 30 × 10^6^ colony forming unit (CFU) of 100 *μ*L with the help of a syringe. A coated suture was then placed in one incision, whereas an uncoated suture was placed in the other incision. A discontinuous suturing was done to close the incision site. The same procedure was carried with the mice using* P. aeruginosa* (50 × 10^6^ CFU) of 100 *μ*L for inoculation. The entire experiment was performed in triplicate using sterilized instruments. The sutured incision sites were covered with surgical tape for two days. After two days, sutures from both sides of mice were taken out and placed in separate 1.5 mL centrifuge tubes containing 100 *μ*L PBS solution, the sutures were placed on the Petri dishes containing nutrient agar and placed in an incubator at 37°C overnight.

### 2.11. Statistical Analysis

All the quantitative data were expressed as mean value with standard deviation. The statistical analyses of the results were done by using *t*-test in Graph Pad Prism 6.0 software. The values that were *p* < 0.05 were considered statistically significant value.

## 3. Results and Discussion

### 3.1. Scanning Electron Microscopy (SEM)

**Figure 1:**
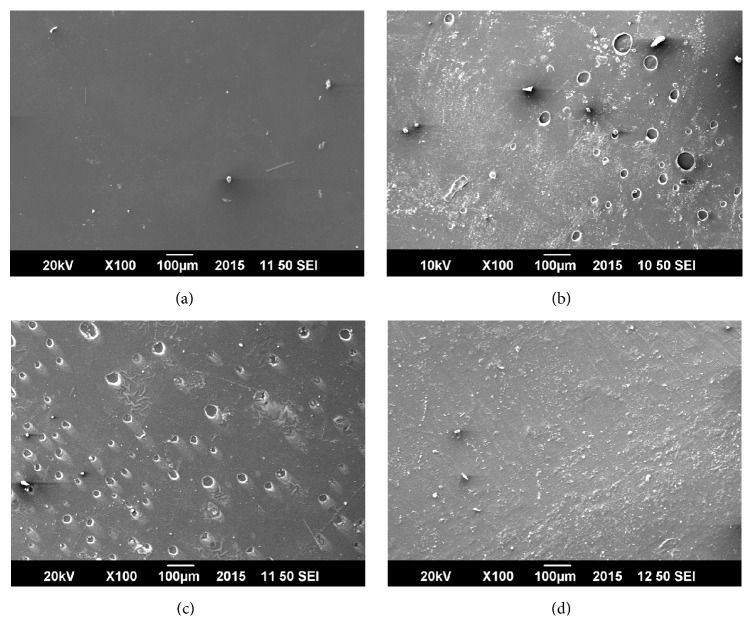
SEM images of* Aloe vera*/polymer films. (a) Film with 5%* Aloe vera* concentration at 20 kV and ×100 magnification. (b) Film with 10% concentration at 10 kV and ×100 magnification. (c) Film with 15% at 20 kV and ×100 magnification and (d) with 20% concentration at 20 kV and ×100 magnification.

The surface morphology of different films was assessed by SEM which has been demonstrated in Figure 1. The SEM images showed the aggregates of* Aloe vera* dispersed on the surface of films which contributed to the film surface roughness. Similar results have been reported by Pereira et al., while studying the properties of alginate based* Aloe vera* films [8].

### 3.2. Fourier Transform Infrared (FTIR)

**Figure 2:**
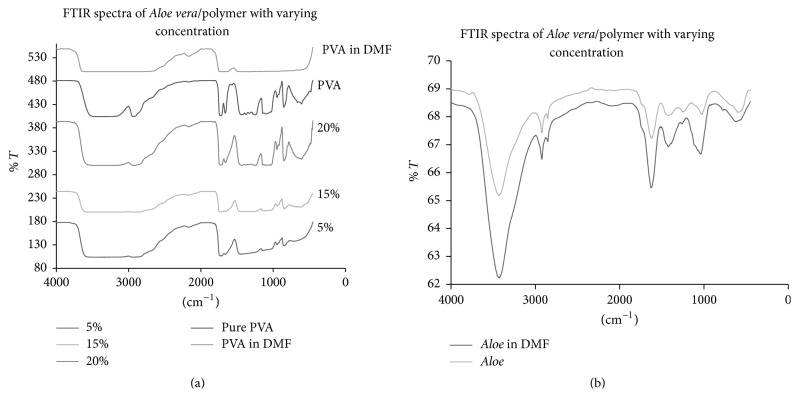
FTIR results of (a) PVA film and* Aloe vera*/polymer films with 5%, 15%, and 20% and (b) results of pure* Aloe* and* Aloe* incubated in DMF.

FTIR analysis was performed to identify the nature of linkages between PVA and* Aloe vera*. The FTIR spectra of pure* Aloe vera*, PVA,* Aloe vera* in DMF, and* Aloe vera*/PVA films with varying concentrations have been shown in Figure 2. The peak that appeared between 3500 cm^−1^ and 3200 cm^−1^ in all films indicates the presence of hydroxyl group (OH) [24]. The absorption band between 3000 cm^−1^ and 2800 cm^−1^ centered at 2932.68 cm^−1^ in 5%* Aloe vera*/PVA and 2926 cm^−1^ in 20%* Aloe vera*/PVA. Both peaks had shifted from 2922 cm^−1^; this was a characteristic of asymmetric stretching of CH_2_ groups [25]. The shift indicated the intermolecular interactions at these functional groups in* Aloe vera* and PVA. The peaks obtained at the range of 1720 cm^−1^ to 1710 cm^−1^ correspond to the stretching of C=O group which indicated the presence of carbonyl compounds in* Aloe vera*. The presence of C-O-C (phenol ether) group was indicated by the bands located at 1036 cm^−1^ in films having 20%* Aloe vera*/PVA concentration. The peak in pure* Aloe vera* at 1075 cm^−1^ [25] was shifted to 1036 cm^−1^ indicating the presence of C-N functional groups in the films; the shift observed in the peak can be attributed to interactions between amine groups and hydroxyl groups of* Aloe vera* and PVA, respectively [26]. The absorption band 1460 cm^−1^ to 1410 cm^−1^ appeared in all concentrations of* Aloe vera*/PVA films, hence representing symmetric stretching vibrations of COOH groups in films [26]. The broad peak at 1150 cm^−1^ to 1130 cm^−1^ could indicate either (C-O) stretching vibrations in films with concentrations of 5% and 15%. The absorption peaks obtained at 860 cm^−1^ to 840 cm^−1^ correspond to rocking vibrations of CH_2_ bonds in PVA [27]. The bending of C-H alkyl groups present in* Aloe vera* and PVA at a peak range of 950 cm^−1^ to 940 cm^−1^ can easily be seen in FTIR results. A new peak at 2171.18 cm^−1^ in 5%, 2167.69 cm^−1^ in 15%, and 2168 cm^−1^ in 20%* Aloe vera*/PVA film indicates the occurrence of interactions between CH group of PVA with CH group of* Aloe vera*. The band at 1660 cm^−1^ and 1264 cm^−1^ in 20%* Aloe vera*/PVA film demonstrated the interaction between hydrogen groups and C-O-C of PVA and C=O and C-O-C groups of* Aloe vera* [25, 28, 29].

The occurrence of peaks of COOH, C-H, C-O-C, NH_2_, and OH shows that the pharmacologically active compounds of* Aloe vera* such as anthraquinones, saponins, and polysaccharides are still in their active form. This can be further correlated with antibacterial activity which confirmed that the active components are still intact and are not affected through interactions with PVA. Thus, the stability of pharmacologically active moiety of* Aloe* after preparation in DMF and loading in PVA films has been confirmed.

### 3.3. Antimicrobial Testing Results of Films

**Figure 3:**
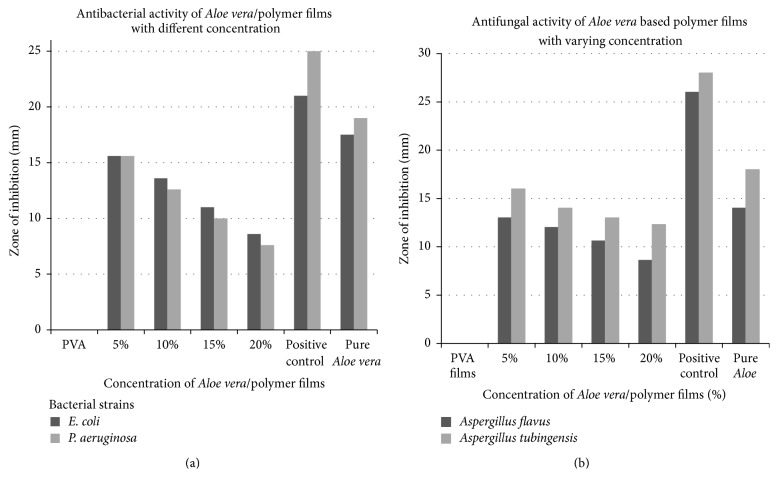
Graphical representation of antibacterial (a) and antifungal (b) activity of different concentrations of* Aloe vera*/polymer films. *y*-axis shows zones in mm while *x*-axis shows varying concentration of* Aloe vera*/polymer films.

**Figure 4:**
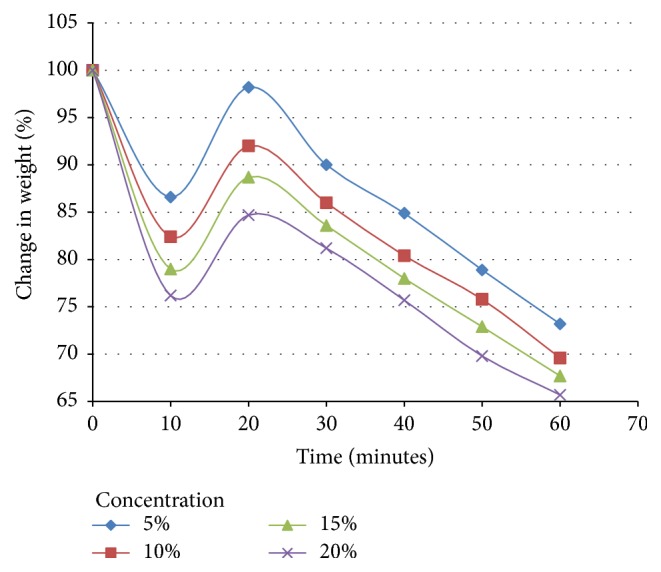
Degradation profile of* Aloe vera*/polymer films with varying concentration. Time in minutes is shown on *x*-axis and % change in weight is shown on *y*-axis.

**Figure 5:**
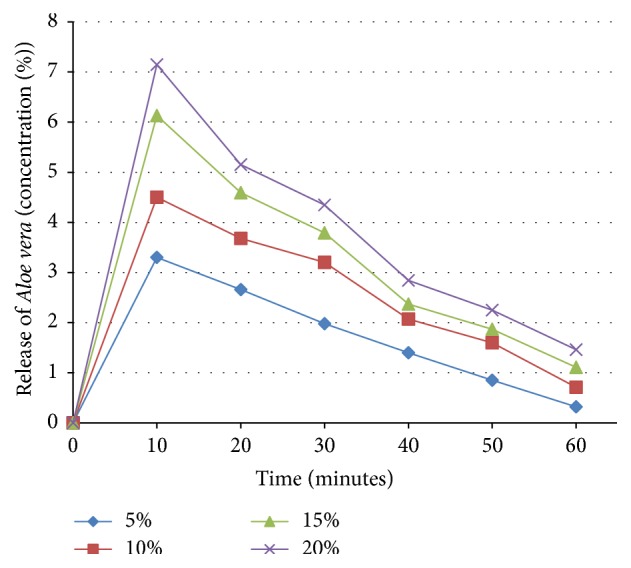
*Aloe vera* release profile of* Aloe vera*/polymer films with different concentrations. *x*-axis shows time in minutes and *y*-axis shows release of* Aloe vera* concentration in %.

The* Aloe vera*/PVA films when positioned on bacterial and fungal inoculated plates gave zones of inhibition which were recorded after 24 h of positioning the films (Figure 3). All films demonstrated the antimicrobial activity due to the release of* Aloe vera* from the surface of the films. The maximum activity was indicated by 5%* Aloe vera*/PVA combination. The potential reason could be the presence of a lower number of interactions between* Aloe vera* and PVA; because of lower concentrations of* Aloe* gel, they were not chemically bound to each other thus keeping the components and their respective functional groups of* Aloe vera* chemically active against microbial activity as shown by FTIR results. Increased levels (10%, 15%, and 20%) of* Aloe vera* in the PVA blend lead to the increased interactions between pharmacologically active components of* Aloe vera* and PVA which causes the shift in FTIR peak (Figure 2); thus such interactions of* Aloe vera*/PVA may influence antimicrobial activity. Renisheya Joy Jeba Malar et al., demonstrated the antimicrobial activity of DMSO extracts of* Aloe vera* gel against human pathogens and highest zone of inhibition (13 mm) against* E. coli* was recorded [30]. In another study, the zone of inhibition against* E. coli*,* P. aeruginosa*, and* Aspergillus flavus* was recorded as 15 mm, 20 mm, and 15 mm, respectively [31]. In current research, the mean zone of inhibition is 15 mm for both* E. coli* and* P. aeruginosa* and 16 mm for* Aspergillus tubingensis* (Figures 4 and 5) while for pure* Aloe vera* the zone was 19 mm and no zone was recorded against PVA films. Thus it can be concluded that blend of* Aloe vera* and PVA has not much affected the antimicrobial activity of* Aloe vera* and antimicrobial activity of* Aloe vera* was maintained in blend form.

### 3.4. Degradation of* Aloe vera* Release Profile Test Results

The degradation profile of* Aloe vera*/PVA composite was evaluated by recording weight loss at predetermined time points (Figure 4). The degradation profile was divided into three stages; during first 10 minutes a sudden loss of weight was observed due to the initial burst release of* Aloe vera*, followed by sudden increase in the weight of the films (Figure 4) because of the absorption of buffer solution by the PVA. PVA, when exposed to aqueous media, absorbs the liquid and swells, resulting in an increase in weight; later it becomes solvated and starts losing mass [32]. However, after 30 minutes, the weight loss by the films became linear. The rate of swelling of the PVA films after the initial burst decreased with the increase in the ratio of the* Aloe* component of the films. This was due to the fact that, with the increase in the* Aloe vera* concentration of PVA based films, absorption of the liquid medium by the PVA decreased [33].

The initial burst release followed by slow surface release of* Aloe vera* from the polymer based* Aloe vera* films was observed (Figure 5). An initial burst release of* Aloe vera* from the surface of the films was detected during the first 10 minutes. This may be attributed to the presence of aggregates of* Aloe vera* components on the film surface (verified later by SEM images of the films). The aggregates of* Aloe vera* over the surface of the films were observed causing the initial burst release. Later, the amount of* Aloe vera* released from the surface decreased because of entrapment of* Aloe vera* in PVA mass. During first 10 minutes* Aloe vera* was released only by diffusion from the surface while after 20 minutes the degradation of* Aloe vera*/polymer film also contributes to the release of* Aloe vera* [34]. The release profile of all the concentrations, that is, 5%, 10%, 15%, and 20%, showed the same behavior but with the increase in concentration from 5% to 20% greater initial burst release was observed which is due to the increased amount of* Aloe vera*. Moreover, increased concentration resulted in decreased* Aloe* release from the surface in the later stages because increasing the* Aloe* amount lowers the rate of diffusion of* Aloe* from the surface [35].

An accelerated degradation and release study is performed for short period of time because, keeping the application in mind, the chance of infections occurring is greater at initial stages. The* Aloe vera*/polymer composite film is flexible and can easily be placed on body surfaces, hence making it an ideal candidate for wound healing devices. The initial* Aloe vera* release from the surface is intended to be used as an antimicrobial so as to prevent the entry and proliferation of the microbes into the wound area [36]. Also, slow release marks the potential for an ideal microbial-free environment for wound healing [36].

### 3.5. Coating of the Sutures

The dry weight of the sutures before dipping into the coating solution was 0.045 g and after dipping into the coating solution was increased to 0.075 g. This increase in weight demonstrated the coating of the suture with the coating material.

### 3.6. *In Vitro* Testing of Coated Suture

**Figure 6:**
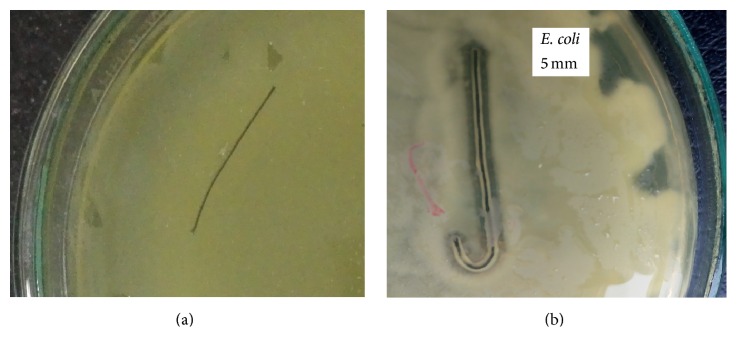
*In vitro* testing against* E. coli* of (a) uncoated suture and (b) coated suture.

The zones of inhibitions against both the bacterial strains (*E. coli* and* P. aeruginosa*) were evaluated with coated and uncoated sutures (Figure 6). The results were compared with uncoated sutures which demonstrated no zone of inhibition, using a paired *t*-test (Table 1).

The zone of inhibition with* E. coli* was 4.6 ± 0.577 mm (mean of three triplicates), *p* value = 0.0051, while with* P. aeruginosa* it was 3.16 ± 0.28 mm (mean of three triplicates), *p* value < 0.0028.

### 3.7. *In Vivo* Testing of Coated Sutures

**Figure 7:**
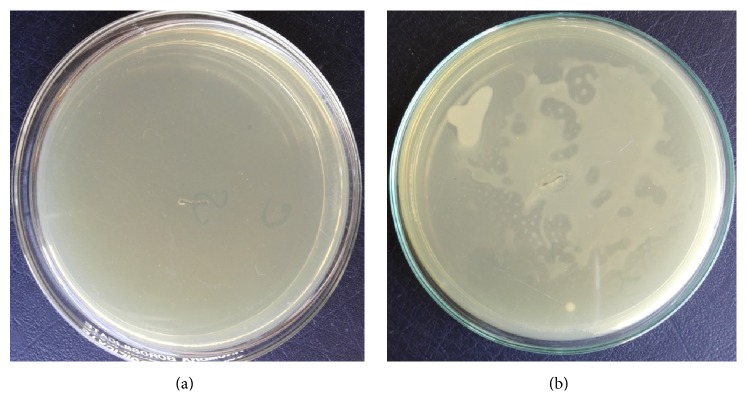
*In vivo* colonization of* E. coli* with coated and uncoated suture (a) shows the results of* in vivo* antibacterial activity with coated suture while (b) shows the* in vivo* antibacterial results with uncoated sutures.

The silk sutures coated with* Aloe vera*/polymer coatings showed significant reduction in microbial colonization by* E. coli* and* P. aeruginosa* in mice models (Table 2). The coated sutures demonstrated reduction in* E. coli* to about 97% (*p* < 0.0001) and 80% with* P. aeruginosa* (*p* < 0.0001) (Figure 7).

In this present study, silk sutures coated with* Aloe vera*/polymer coating exhibited substantial zone of inhibitions against* E. coli* and* P. aeruginosa in vitro* because of the pharmacologically active components present in* Aloe vera* such as anthraquinones which remain active after being blended with PVA. Coated sutures showed results against bacterial strains while no inhibition zones were observed with uncoated sutures. For* in vivo* studies, mice models were used in which control and test sutures were used in the same animal and the incision site was inoculated with a known number of bacteria to evaluate the effectiveness of the coated sutures. The results of both* in vivo* and* in vitro* studies along with FTIR confirmed that the active components that are responsible for antimicrobial activity present in* Aloe vera* still remain in their active form.

Sutures with* Aloe vera*/polymer coating illustrated noticeable reduction in the growth of the* P. aeruginosa* and even greater reduction against* E. coli*. The test results of the* in vivo* and* in vitro* investigations suggested that sutures with* Aloe vera*/polymer coating are bactericidal. It was verified by calculating the bacterial colony count at incision site which was reduced in case of coated sutures; this shows that the* Aloe vera*/PVA composite may be used as a suture coating that has the potential to prevent the spread of infections during surgical procedures.

## 4. Conclusion

The biocompatibility and biodegradative properties of PVA have been combined with the intrinsic bactericidal properties of* Aloe vera*. The composition was screened for antimicrobial activity against bacterial and fungal strains, that is,* E. coli*,* P. aeruginosa*,* Aspergillus flavus*, and* Aspergillus tubingensis*, respectively. The polymeric films with lowest concentration (5%) of* Aloe vera* illustrated the best results with regard to antimicrobial activity against all the strains. Commercially available sutures were coated with* Aloe vera*/PVA solution and tested for antimicrobial activity in* in vitro* and* in vivo* systems. These coated sutures illustrated a potential for antibacterial/antifungal coatings in commercial surgical sutures that can play a role in preventing infections at surgical sites. Biocompatibility tests and clinical trials need to be conducted to better ascertain the potential of this* Aloe*/polymer composite as an option for use in surgical procedures as a suture coating, as part of prophylactic measures to prevent surgical infections.

## Figures and Tables

**Table 1:** *In vitro* testing of coated and uncoated sutures against *E. coli *and *P. aeruginosa*.

Bacteria	Zone of inhibition (mm)	
	Coated suture	Uncoated suture
*E. coli*	4.6 ± 0.577	0 ± 0
*P. aeruginosa*	3.16 ± 0.288	0 ± 0

**Table 2:** *In vivo* bacterial colonization of suture with coated material.

Bacterial strains	Log CFU/explanted^a^	% kill bacteria relative to inoculum introduced	*p* value^b^
*E. coli*			
With coated material	03	97	<0.0001
Without coated material	>300	NA	
*P. aeruginosa*			
With coated material	11	80	<0.0001
Without coated material	>300	NA	

^a^Average of three animals.

^b^Paired  *t*-test.

NA: not applicable.
